# RNA-Seq and Microarrays Analyses Reveal Global Differential Transcriptomes of *Mesorhizobium huakuii* 7653R between Bacteroids and Free-Living Cells

**DOI:** 10.1371/journal.pone.0093626

**Published:** 2014-04-02

**Authors:** Jieli Peng, Baohai Hao, Liu Liu, Shanming Wang, Binguang Ma, Yi Yang, Fuli Xie, Youguo Li

**Affiliations:** 1 State Key Laboratory of Agricultural Microbiology, Huazhong Agricultural University, Wuhan, Hubei, P. R. China; 2 Center for Bioinformatics, School of Life Science and Technology, Huazhong Agricultural University, Wuhan, Hubei, P. R. China; Max F. Perutz Laboratories, Austria

## Abstract

*Mesorhizobium huakuii* 7653R occurs either in nitrogen-fixing symbiosis with its host plant, *Astragalus sinicus*, or free-living in the soil. The *M. huakuii* 7653R genome has recently been sequenced. To better understand the complex biochemical and developmental changes that occur in 7653R during bacteroid development, RNA-Seq and Microarrays were used to investigate the differential transcriptomes of 7653R bacteroids and free-living cells. The two approaches identified several thousand differentially expressed genes. The most prominent up-regulation occurred in the symbiosis plasmids, meanwhile gene expression is concentrated to a set of genes (clusters) in bacteroids to fulfill corresponding functional requirements. The results suggested that the main energy metabolism is active while fatty acid metabolism is inactive in bacteroid and that most of genes relevant to cell cycle are down-regulated accordingly. For a global analysis, we reconstructed a protein-protein interaction (PPI) network for 7653R and integrated gene expression data into the network using Cytoscape. A highly inter-connected subnetwork, with function enrichment for nitrogen fixation, was found, and a set of hubs and previously uncharacterized genes participating in nitrogen fixation were identified. The results described here provide a broader biological landscape and novel insights that elucidate rhizobial bacteroid differentiation, nitrogen fixation and related novel gene functions.

## Introduction

Rhizobia are specific soil bacteria, which can establish an intricate symbiotic relationship with Legumes. This interaction results in the formation of specialized root structures called nodules [1]. In the indeterminate nodules of the Inverted Repeat Lacking Clade (IRLC) legumes [2] (e.g. *Medicago*, *Pisum*, and *Astragalus sinicus*), bacteria undergo dramatic changes in size, shape, and DNA content to become terminally differentiated bacteroids [3]. The host plant provides rhizobia with dicarboxylic acids as a source of carbon, energy, and reductant [4] and ammonium (via N_2_ reduction that occurs in these bacteroids) is secreted back to the plant. This biological process serves as a natural form of fertilization and has significant potential for use in sustainable agricultural programs, especially if optimized. Thus, Rhizobium-legume symbiosis has been the subject of much study for decades.

*Mesorhizobium huakuii* 7653R is a α-proteobacterium that occurs either in a nitrogen-fixing symbiosis with its host plant, *A. sinicus*, or free-living in the soil. *M. huakuii* 7653R is a narrow-host-range rhizobium that induces indeterminate-type nitrogen-fixing nodules on the roots of *A. sinicus*, which is an economically important forage and green manure growing in winter throughout Eastern Asia. The whole-genome sequence of *M. huakuii* 7653R has been recently completed (http://www.ncbi.nlm.nih.gov/genome/11322). Transcriptome analysis has always played a central role for unraveling the complexity of gene regulation in the field of functional genomics. Transcriptome profiling is traditionally done using either real time quantitative PCR to examine a few genes [5], or Microarrays to examine genome-wide transcriptional activity [6]. As the genome sequences of several rhizobia are readily available [7]–[10], it is possible to use Microarrays to study complex host-microbe interaction. Formation of determinate nodules in the *Bradyrhizobium japonicum*-*Glycine ma*x symbiosis [11], [12] and the *Mesorhizobium loti-Lotus japonicus symbiosis* [13] have been investigated with Microarrays. Indeterminate-type nodule formation, as occurs in *Sinorhizobium meliloti*-*Medicago sativa* symbiosis [14] or *Rhizobium/pea-vetch* symbiosis [15] have been studied with Microarrays as well. These studies have provided significant insight into bacteroid function and development. Most of the publications focus on the analysis of individual metabolic pathways, a global perspective of the interaction mechanism is still limited. Therefore, it is difficult to fully understand the complex metabolic changes and gene networks that occur in bacteroids relative to free-living cells.

Recently, as a result of the decreased cost of next generation sequencing technologies [16], RNA sequencing (RNA-Seq) is becoming the method of choice in transcriptome studies[17]. However, study of the bacterial transcriptomes has not progressed as quickly as the study of eukaryotic transcriptomes [18], [19] due to the lack of mRNA polyA tails in bacteria, which prevents specific targeting of the mRNA versus the much larger rRNA pool. Therefore, the application of RNA-Seq in prokaryotes requires additional steps in the RNA preparation procedure to increase the relative abundance of mRNA and new methods to replace the poly (T)-primed approach in cDNA synthesis. Yoder-Himes et al. (2009) successfully resolved these limitations and subsequently used RNA-Seq to investigate the bacterial transcriptome in *Burkholderia cenocepacia* [20]. However, few have investigated the *Rhizobium* transcriptome using this method, until recently in *Sinorhizobium sp.* NGR234 bacteroids in determinate nodules of *Vigna unguiculata* and indeterminate nodules of *Leucaena leucocephala* have been studied with RNA-Seq [21]. To date, Microarrays remains a useful and accurate tool for measuring expression levels, and RNA-Seq complements and extends data obtained from Microarrays. Studies have demonstrated that RNA-Seq and Microarrays do in fact complement each other in transcriptome profiling. There is an obvious advantage to applying multiple transcriptome profiling methods, providing a comprehensive picture of a transcriptome, rather than relying solely on one method [22]. To better understand the complex biochemical and developmental changes that occur in *M. huakuii* 7653R during bacteroid development, we performed genome-wide transcriptome profiling by using a combination RNA-Seq and Microarrays technology. As a result, we have provided a broader biological landscape and novel insight that advances our understanding of rhizobium response to alterations in the symbiotic environment, and what physiological changes occur during bacteroid differentiation.

## Materials and Methods

### Bacterial strains and growth conditions

*M. huakuii* 7653R was grown at 28°C in TY medium (5 g/liter tryptone, 3 g/liter yeast extract, and 1.3 g/liter CaCl_2_·6H_2_O[pH 7.0])[23]. To isolate RNA from free-living cells, *M. huakuii* 7653R was grown in 100 mL of medium up to an optical density at 600 nm of 1 to 2. Cultured cells were harvested by centrifugation at 12 000 g at 4°C for 1 min. The pellets were frozen in liquid nitrogen and stored at −80°C until RNA was isolated.

### Plant growth, inoculation, and nodules harvest

*A. sinicus* seeds were sterilized, germinated, planted, and inoculated with *M. huakuii* 7653R strains as previously described[24]. At 32 days post-inoculation, the nodules of plants were harvested, immediately frozen in liquid nitrogen, and stored at −80°C until RNA was isolated.

### RNA extraction and bacteroids RNA isolation

Total RNA was extracted using TRIzol Reagent (Invitrogen, Carlsbad, CA) according to the manufacturer's instruction. To remove contaminating genomic DNA, approximately 1 μg of RNA was treated with 1 U of DNaseI (Fermentas, Burlington, VT, Canada) at 37 °C for 20−30 min. These reactions were terminated by the addition of 1 μL of 25 mM EDTA (Invitrogen) and incubation at 65°C for 10 min. To isolate bacteroid RNA, total nodules RNA passed though MICROB*Enrich* kit (Ambion) according to the manufacturer's instruction. This step was intended to remove plant mRNAs and also 18S and 28S rRNAs. However, as it was necessary to reduce variation resulting from sample preparations, RNA substrates were extracted from the same phase (OD600 of 1 to 2 free-living cells and 32 days post-inoculation nodules) and were carried out using independently isolated RNA preparations from independent cultures and set of plants.

### RNA-Seq experimental design, libraries construction and sequencing

RNA-Seq libraries were prepared for each sample following standard protocols (Illumina, San Diego, CA). Briefly, mRNA was isolated from total bacteroids RNA using MICROB*Express* kit (Ambion). Isolated mRNA was fragmented using divalent cations under increasing temperatures followed by ethanol precipitation. The fragmented mRNA was reverse-transcribed into cDNA using Superscript III and random primers. The cDNA was end-repaired to create blunt-ended fragments, and an ‘A’ base was ligated to the 3′ ends of the fragments to create an ‘A’ base overhang. Adapters with a ‘T’ base overhang were ligated to each end of the cDNA fragments. The ligated fragments were run on an agarose gel, and a thin slice of DNA fragments corresponding to approximately 200 bp was excised and purified from the gel. The purified size-selected fragments were enriched by PCR to generate the final library fragments. Sequencing reactions were carried out on the Illumina HiSeq 2000. The read length was 90 bp, paired end sequencing method was used. RNA-Seq experiments produced 1.07 Gb and 1.13 Gb sequences for the free-living and bacteroids, respectively. Total mapped reads to genome is 94.93% and 17.31% for the free-living and bacteroids, respectively. Around 200 Mb sequences from the bacteroids' sample were uniquely mapped to M. huakuii 7653R genome (6.6 Mb).

### The experimental design and gene expression analysis of Microarrays

Microarrays oligonucleotides were designed according to the 7,453 annotated open reading frames (ORFs) of *M. huakuii* 7653R. For each gene, three probes were designed and each duplicated three times. Microarrays were prepared for each sample following the User's Guide (Roche NimbleGen, Madison, WI). Briefly, total RNA was added a poly(A) tail using Poly(A) Polymerase. Poly(A) RNA was reverse transcribed with a T7 promoter site Oligo dT primer to synthesize a first-strand cDNA using SuperScript II. T7 promoter site was completed by a second strand cDNA synthesis. The RNA Polymerase driven in vitro transcription converts the double-stranded cDNA with T7 RNA Polymerase promoter site into the final product, amplified-antisense RNA (aRNA). 5 μg cRNA was reverse transcribed with random primer by using CbcScript, then it was purified by using NucleoSpin Extract II Kit (MN). Double-stranded cDNA was primed with random primer Cy3 or Cy5-dCTP (GE Healthcare) using Klenow enzyme. At the completion of the procedure, the quality of the purified Cy3 or Cy5 labeled cDNA was assessed by using a spectrophotometer. Pairs of samples designed for hybridization to the same array were labeled in parallel. Bacteroids samples were labeled with Cy3 and free-living cells samples were labeled with Cy5. Cy-labeled cDNA was hybridized with high-density, 70-mer oligonucleotide Microarrays slide mixer in a Roche NimbleGen Hybridization System 12 for 14-16 h at 42°C. The hybridized slides were washed and scanned by using Roche-NimbleGen MS200. A total of three independent biological replicates were designed for each condition, which included a dye-swap for each replicate, resulting in a total of 6 slides for the experimental condition.

### Statistical analysis of RNA-Seq and microarrays data

Microarrays images and intensity data were uploaded to NimbleScan (version:2.6.0.0) to analyze data extracted from the image. Briefly,a tif image was uploaded into NimbleScan along with NimbleGen design files, which describe both probe identities and locations, to generate a tab-delimited report with probe identities and feature signal intensities. At the completion of the procedure, data were present in an appropriate format for normalization and comparative analysis. Microarrays images with uniformity hybridization signal and the area of scratches, bubbles and other dust no more than 5% were analyzed. The Alignment Oligo is a mixture of Cy3- and Cy5-labeled 48 mer oligonucleotides that hybridize to alignment features on NimbleGen arrays. Twelve Sample Tracking Controls (STCs) were used. Each STC is a Cy3-labeled 48 mer oligonucleotide. When a unique STC was added to each sample before hybridization to a multiplex array, the STC could be used to confirm that the correct sample was hybridized to each array. This control hybridized to probes on the Microarrays and enabled to confirm the sample identity on each array and the integrity of the experiment. STC probes were placed as repeating sets of 20 along the perimeter of each array and as two 4 × 5 blocks in the upper left corner and in the center of the array. The data files were normalized for correcting the signal value by RMA (Robust Multichip Analysis) and then analyzed by SAM (Significance Analysis of Microarrays) [25].

RNA-Seq images generated by sequencers were converted by base calling into nucleotide sequences, which were called raw data or raw reads. Reads with adaptors, unknown nucleotides larger than 10% and low quality (more than half of the bases' qualities are less than 5) were removed, and the clean reads were then obtained. Clean reads were mapped to M. huakuii 7653R genome and gene sequences respectively using SOAP2[26]. Mismatches of no more than 5 bases were allowed in the alignment. To eliminate the influence of different gene length and sequencing discrepancy on the calculation of gene expression, the RPKM (reads per kilobase per million mapped reads) method was used to calculate gene expression level [27]. Genes with the ratio of RPKMs of the two samples above 2, Benjamini FDR (False Discovery Rate) ≤0.001 were defined as the differentially expressed genes between two samples.

Differentially expressed genes of statistical significance from both RNA-Seq and Microarrays data were determined by applying a threshold of p/q value ≤ 0.05 (5%) and absolute fold-change ≥ 2. In order to identify those genes with a relevant role in bacteroid metabolism and, in turn, form a set of genes that serve as a benchmark for computational assessment, we took into account both sources of data following an integrative rather than a selective strategy.

### PPI network reconstruction and analyses

Information found in PPIs databases supports the construction of interaction networks. Here, we elucidated the homologous protein-protein interactions network through the concepts of orthologous conservation (or interologs) [27]. In this work, *M. huakuii* bv *loti* MAFF303099[28] and *Escherichia coli* [29] PPI network databases were used as references, and pairwise genome comparison for bidirectional best hits (BBH) [30] was adopted to acquire high-quality PPI network of *M. huakuii* 7653R. Briefly, in pairwise genome comparison for BBH, we set the e-value < 1e-5. Identity≥0.3, coverage≥0.7. The workflow is illustrated in Figure S1. In total, 10 306 pairs of non-redundant PPIs were isolated (Table S1). Cytoscape[31] was used for network visualization and analysis. jActiveModules[32] plugin was used to search subnetworks of differentially expressed genes and BiNGO[33] plugin was used to find the up-regulated GO terms and to display the functional enrichment of the GO hierarchy where the up-regulated terms were from as a graph in Cytoscape. The CtrA subnetwork was dissected from its first two neighbors. Briefly, first found the first neighbors of selected Nodes (CtrA) that we can get several genes, second found first neighbors of these genes.

### RNA-Seq and microarrays data accession number

RNA-Seq and Microarrays data were submitted to the Gene Expression Omnibus (http://www.ncbi.nlm.nih.gov/geo/) under GEO accession no. GSE48105. The accession numbers of RNA-Seq and Microarrays data are GSE48103 and GSE48090, respectively.

## Results and Discussion

In this study, we analyzed the global gene expression profiles that are present during bacteroid differentiation. Therefore, it was critical to prepare sufficient and purified RNA samples. As bacteroids are enclosed by a peribacteroid membrane of plant origin [34], it is difficult to extract bacteroids RNA from nodules, particularly, from the nodules of *A. sinicus* induced by *M. huakuii* 7653R because of their smaller size.

With traditional protocols [15], [35], the bacteroids are usually isolated from nodules via physical separation, prior to bacteroid RNA extraction. It is inherently difficult to preserve bacteroid structure and thus prevent contamination by plant materials. This isolation process also takes longer to accomplish, thereby making it difficult to investigate real-time bacteroid gene expression. Traditional isolation procedures are somewhat complicated and also require large quantities of nodules; even still the resulting RNA is often of unsatisfactory quality. Since RNA-Seq requires large quantities of high-quality total RNA, we isolated the total RNA directly from the freshly harvested nodules immediately using a TRIzol protocol. We then used a MICROB*Enrich* kit to enrich bacterial RNA from the mixtures of plant and bacterial RNA samples. Our subsequent use of a MICROB*Express* kit resulted in obtaining large quantities of high-quality targeted bacteroid mRNA for use in transcriptome analysis.

### 1 Transcriptome overall analysis based on RNA-Seq and Microarrays

#### 1.1 Global gene expression profile

**Figure 1:**
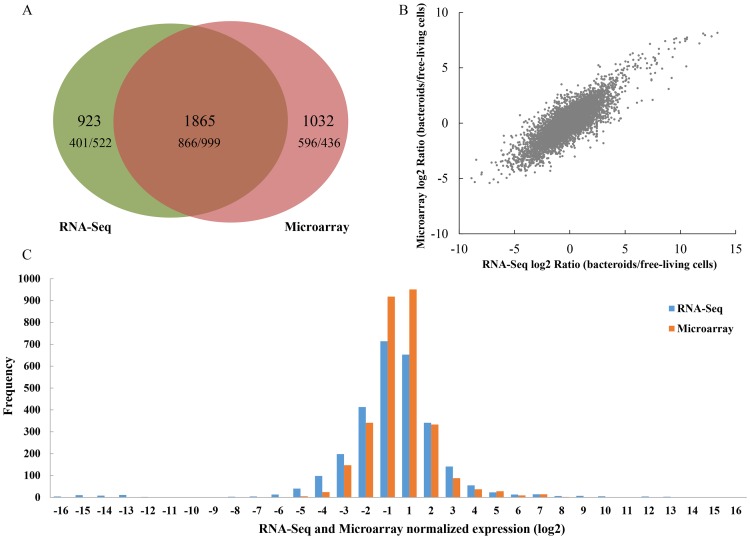
Schematic view of data from RNA-Seq and Microarrays. A: Venn diagram summarizing the overlap between differentially expressed genes from RNA-Seq (*left* circle) and Microarrays (*right* circle). The number of genes called by both methods is indicated by the overlap between the two circles. It also contains the numbers of up/down-regulated genes for each set in the Venn-Diagram. B: Comparison and correlations analyses of differentially expressed genes in RNA-Seq and Microarrays. The plot shows log2 ratios (bacteroids/free-living cells) of expressed genes in RNA-Seq (*x* axis) and Microarrays (*y* axis) (7043 genes in total). Pearson's correlation coefficient (r) is indicated for each comparison. C: Distribution of differentially expressed genes detected by RNA-Seq and Microarrays. Genes detected by RNA-Seq are shown with blue bars. Genes detected by Microarrays are shown with orange bars.

In total 6967 and 6662 expressed genes were detected in *M. huakuii* 7653R during symbiosis through RNA-Seq and Microarray, respectively. Among them, 2788 (RNA-Seq) and 2897 (Microarrays) genes were differentially expressed (|log2| ≥ 1, p/q value <0.05) in bacteroids compared with free-living cells. For the differentially expressed genes, 1267 genes were up-regulated and 1521 genes were down-regulated (as determined by RNA-Seq), while 1462 genes were up-regulated and 1435 genes were down-regulated (as determined by Microarrays). Any overlap of differentially expressed genes as identified by these methods is presented in Figure 1A. For the overlapping 1865 genes, the proportions of up- and down-regulation in plasmid and chromosome were calculated (Table 1). An overall trend showed that the plasmid genes were up-regulated, while chromosomal genes were down-regulated under symbiosis. These data suggest that a significant number of plasmid genes are required for nitrogen fixation, whereas housekeeping genes, those essential for active growth are located primarily on the chromosome.

**Table 1:** The proportions of differentially expressed genes in plasmid and chromosome of *M. huakuii* 7653R during symbiosis.

Category	Number of genes	Number of up-regulated genes	Percentage^a^	Number of down-regulated genes	Percentage^a^
pMHa	257	59	22.96%	18	7.00%
pMHb	447	105	23.49%	26	5.82%
Chromosome	6562	702	10.70%	955	14.56%
Total	7266	866	11.92%	999	13.75%

a The percentage represents the proportion of the up- or down-regulated genes to the total number of genes in the plasmids and chromosome, respectively.

Total expressions of differentially expressed genes were calculated using the expression levels of 2784 and 2750 genes (RNA-Seq) in free-living cells and bacteroids, respectively. There had several genes only been tested in one condition, accordingly the numbers (2784 and 2750) were less than the total differentially expressed genes (2889) in RNA-Seq. This analysis indicated that 80% of the total expression amount is attributed to 506 and 365 genes, respectively. Following the assignment of these genes to functional categories and calculation the distribution of genes among functional categories (Figure S2), from the percentage of each category, we noticed that most types of functional categories were high in free-living cells, and only the energy production and conversion, replication, recombination and repair and transcription were high in bacteroids.

As such most housekeeping genes are switched off in bacteroids, and those involved in reduction of N_2_ to NH_3_ are up-regulated. These data reflect the fundamental biological difference in that free-living cells have a primary role in maintaining basal metabolism, whereas bacteroids have a primary role in nitrogen fixation. More importantly, the expression amount of the top 10 most highly-expressed genes, which located in plasmids in exception of *MCHK_1372*, in bacteroids (Table S2) accounted for up to 32% of the total expression amount. Meanwhile, these top 10 genes in free-living cells accounted for up to 0.18% of the total expression amount. These suggest that gene expression is concentrated to a set of genes (gene clusters) in bacteroids to fulfill corresponding functional requirements.

#### 1.2 Gene expression comparison and correlation

**Figure 2:**
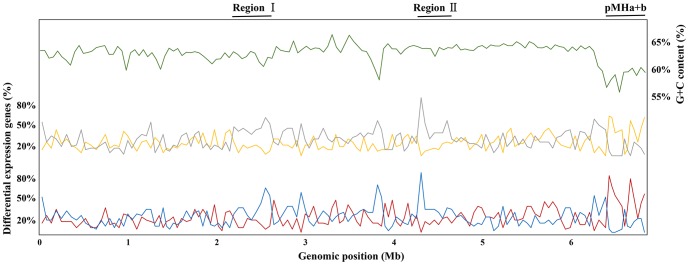
Visualization of global transcriptional profiles as determined by RNA-Seq and Microarrays. The plot shows a summary of RNA-Seq and Microarrays expression data at all regions in bacteroids. Across the entire genome, the percentages of up-regulated genes of RNA-Seq and Microarrays are shown in yellow and red, while those of the down-regulated shown in gray and blue, respectively. The G+C content is in green. The percentage of differentially expressed genes and the GC content were computed with a sliding window of every 50 genes. Note the most prominent up-regulation in the symbiosis plasmids and the most prominent down-regulation in regions I and II in the chromosome.

The expression levels for all the listed (6291) genes as measured by RNA-Seq and Microarrays were examined. Correlation between the two methods was high (Pearson's correlation coefficient *R*  =  0.83), in spite of a compression effect resulting in smaller ratios in Microarrays (Figure 1B). This has been reported previously and is due in part to the limited dynamic range of array experiments [36], [37]. The comparison and distribution of the differentially expressed genes detected by RNA-Seq and Microarrays are presented in Figure 1C. It is apparent that RNA-Seq and Microarrays are consistent with each other in transcriptome profiling ability. Although RNA-Seq had greater accuracy and sensitivity relative to Microarrays, which had a relatively limited dynamic range for the detection of transcript levels due to background, saturation, and spot density and quality [36], [37]. A number of differentially expressed genes with exceptionally high change were detected by RNA-Seq. We listed the top 25 up- (Table S3) and down-regulated (Table S4) genes as called by both RNA-Seq and Microarrays. Most of the top 25 up-regulated genes in RNA-Seq and Microarrays were in parallel (16 genes were identical)and related to nitrogen fixation. In addition, we displayed the global gene expressions by plot visualization (Figure 2). This plot shows the overall location of differentially expressed genes in the chromosome and two plasmids. It was observed that the most prominent up-regulation occurred in the symbiosis plasmids (assigned as region III) and the most prominent down-regulation occurred in chromosome, assigned as regions I and II. The up-regulation regions in the symbiosis plasmids have a remarkable lower GC content. The accumulation of those genes in the symbiotic plasmids was correlated with nitrogen fixation. The two down-regulation regions in the chromosome are correlated with maintaining basal metabolism. The region I has several *fab* genes participating in fatty acid biosynthesis, while the region II encodes many of the flagellar assembly genes. Most genes in these two regions were down-regulated, which reflected the cellular and biological functional changes in the bacteroids to fulfill the symbiotic nitrogen fixation. It is speculated that analyses of the genes within these regions may reveal some novel insights into molecular mechanism of bacteroid differentiation and nitrogen fixation.

#### 1.3 Genetic pathways altered in the bacteroid in response to nitrogen fixation

Even though a variety of sophisticated regulatory mechanisms occur at diverse levels of biological organization [38], we assumed that bacteroid genes significantly up-regulated were closely connected with the functional mechanisms of nitrogen fixation.

**Figure 3:**
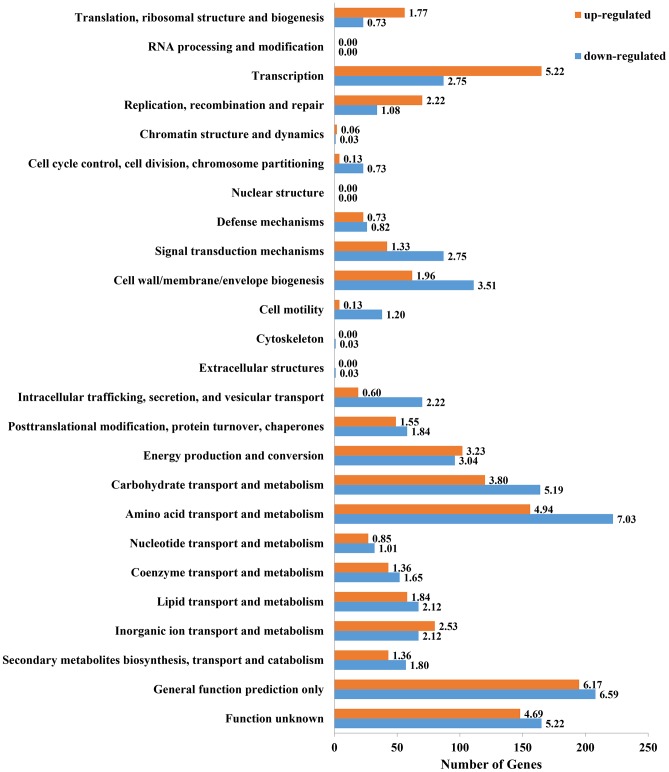
COG functional categories of differentially expressed genes identified by both RNA-Seq and Microarrays. All differentially expressed genes during bacteroids were assigned to the 26 COG functional categories using RPS-BLAST (uncategorized genes not shown). Bars represent the number of up-regulated (orange) and down-regulated (blue) genes in bacteroids compared with free-living cells. The number in each bar represents its up/down-regulated percentage (%).

The assignment of genes to functional categories, as is commonly applied to transcriptome data [39], assists with the identification of differentially regulated pathways, and reduces our dependence on assumptions derived from individuals of differentially expressed genes within large-scale data sets. To survey the role that these genes play in supporting symbiotic nitrogen fixation, we classified them in accordance to the COG functional categories. Approximately 20% of up-regulated genes and 14% of down-regulated genes were classified as uncategorized genes in COG (data not shown). In the functional categories (Figure 3) which were identified as either up- or down-regulated (p/q<0.05), a large proportion of differentially regulated genes were categorized into either: 1) energy production and conversion, 2) amino acid transport and metabolism, 3) carbohydrate transport and metabolism, or 4) transcription. However, most of the down-regulated genes of *M. huakuii* 7653R were associated with cellular processes and signaling. Similar observations have been reported in *S. meliloti* [40] and *R. etli* [41] bacteroids. *S. meliloti* induces indeterminate-type nodules, while *R. etli* forms determinate-type nodules. Hence, the expression profile of bacteroids resembles that of non-growing cells, regardless of whether they are terminally differentiated or not. It is worthy of note that two functional groups, “general function prediction only” and “function unknown” genes, encompassed a large number of differentially expressed genes. This provided novel genetic material, and suggests a need for further investigation.

As there are many known genes important to bacteroid nitrogen fixation, we also identified genes participating in the regulatory mechanisms of nitrogen fixation and nodulation, flagellar assembly, urea cycle, poly-β-hydroxybutyrate (PHB) biosynthesis, protein secretion systems, two-component systems and polysaccharide synthesis (Figure S3). These genes reflect general genetic pathways adapted for use in nitrogen fixation. Some pathways are activated, whereas others are inactivated in bacteroids. For example, the *nif* and *fi*x genes in 7653R bacteroids are all up-regulated, while the *nod* genes are down-regulated or at least retain the same expression level.

#### 1.4 Gene set enrichment analysis

Gene set enrichment analysis (GSEA) [42] was performed to compare bacteroids and free-living cells in this work. GSEA of pathways and genes was performed by using the Category package in Bioconductor ver. 2.6.0 [43]. In this analysis, the gene sets of fewer than 10 genes were excluded. Using a permutation test 10 times, the cutoff of the significance level p values was chosen as 0.01 for the most significant pathways related to WD. Accordingly, the significant pathways were then identified when comparing bacteroids and free-living cells. The dataset has 6646 native features, and after collapsing features into gene symbols, there are 6646 genes. Gene set size filters (min = 15, max = 500) resulted in filtering out 123/197 gene sets, and the remaining 74 gene sets were used in the analysis. In the 74 gene sets, there were 35 and 39 gene sets are up-regulated in free-living cells and bacteroids, respectively. For these gene sets, 14 gene sets are significant at FDR < 25% and significantly enriched at nominal p value < 1% in free-living cells, while 30 gene sets are significant at FDR < 25% and 23 gene sets are significantly enriched at nominal p value < 1% in bacteroids.

In the list of enrichment in free-living cells (Table S5), there were gene sets related to membrane transport and cell motility, growth and death. While in the list of enrichment in bacteroids (Table S6), there were gene sets related to nitrogen metabolism and TCA cycle. GSEA showed that in our transcriptome dataset, free-living cells have a primary role in maintaining basal metabolism, whereas bacteroids have a primary role in nitrogen fixation.

### 2. Central biological metabolic pathways in bacteroids

At the molecular level, symbiotic nitrogen fixation arises as a consequence of the coordinated actions of a variety of genes and metabolites that in turn activate signal transduction cascades within the bacteroid. To survey the role that these differential expression genes played in supporting symbiotic nitrogen fixation, we tested for enriched KEGG pathways. In the list of enriched KEGG pathways (Table S7, Table S8), in the most 25 prominent KEGG pathways in free-living cells and bacteroids, we can see some pathways were different. In free-living cells, there were fatty acid metabolism, cell cycle, flagellar assembly and bacterial chemotaxis, while in bacteroids, there were glycolysis/gluconeogenesis, carbon fixation pathways, pentose phosphate pathway, citrate cycle (TCA cycle) and nitrogen metabolism. These results also support the point that free-living cells have a primary role in maintaining basal metabolism, whereas bacteroids have a primary role in nitrogen fixation.

**Figure 4:**
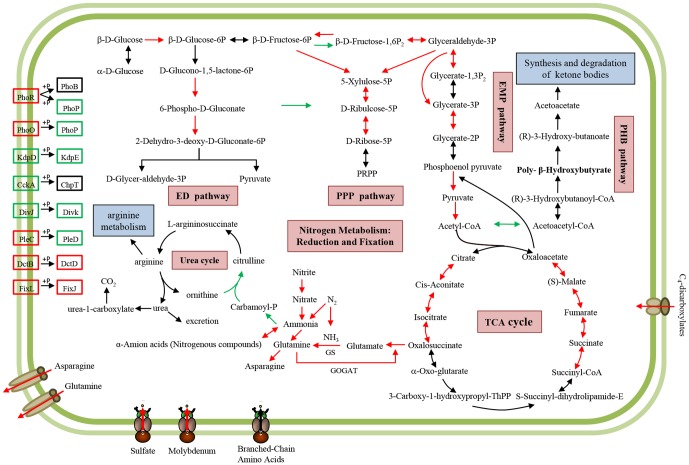
Schematic overview of important KEGG metabolic pathways in bacteroids. Arrows indicate the direction of the metabolism reaction. The enzymes participating in a specific step of reaction were shown in red for up-regulation, green for down-regulation and black for unchanged expression level. Several important differentially expressed genes of two-component systems and transporters (responsible for transportation of glutamine, asparagine, sulfate, molybdenum and C_4_-dicarboxylates) were displayed with the same patterns for direction and color.

To analyze the central metabolism in bacteroids and to visually identify these metabolic reactions, we connected a set of differentially expressed genes (identified via high-throughput analyses) and the enzymes from each metabolic pathway as defined in the KEGG database. A comparative analysis between bacteroid and free-living cell in each pathway allowed us to visualize and highlight any differences. We selected several important KEGG metabolic pathways and illustrated them in a cellular framework (Figure 4) to clearly demonstrate and identify the metabolic pathways activated or suppressed. Overall, C_4_-dicarboxylic acids provided by the host plant were transported into bacteroids and the main energy metabolism pathway remained active, as were ammonium synthesis pathways which supply ammonium to the host plant as a return.

#### 2.1. Glycolysis, gluconeogenesis, Entner-Doudoroff and pentose phosphate pathways

As shown in Figure 4, most of the glycolytic and gluconeogenic enzymes identified were up-regulated. It accords with the report that a common metabolic trait for some rhizobia is the intense activity of gluconeogenesis pathway [44]. This indicated that the gluconeogenesis pathway actively participates in nitrogen fixation. There is evidence that the pentose phosphate pathway also actively participates in nitrogen fixation in *R. etli* [45]. While the pentose phosphate and Entner-Doudoroff pathways coordinately serve as probable major routes for the metabolism of sugars in fast-growing rhizobia [46]. In our results, most of the enzymes in pentose phosphate and Entner-Doudoroff pathways were up-regulated, which is consistent with the needs of the highly energy-dependent symbiotic nitrogen fixation reactions.

#### 2.2. Citric acid cycle

Plants provide rhizobia with C4-dicarboxylic acids such as malate, fumarate, and succinate, which are imported into bacteroids via the dicarboxylate transport system (Dct) [47]. The Dct system of rhizobia consists of a dicarboxylate carrier protein DctA and a two-component regulatory system of DctB and DctD that serve as sensor kinases and transcriptional activators, respectively [48]. In this study, *dctA*, *dctB* and *dctD* genes were up-regulated. As C4-dicarboxylic acids are the sole carbon source to support bacteroid nitrogen fixation and are metabolized exclusively by the tricarboxylic acid (TCA) cycle, it was not surprising to find that *dct* genes were active in bacteroids. Interestingly, a part of the decarboxylating arm of the TCA cycle (involved in citrate synthase, aconitase, isocitrate dehydrogenase, and 2-ketoglutarate dehydrogenase) was transcriptionally up-regulated in bacteroids. Meanwhile the metabolism of malate and succinate was highly up-regulated since α-ketoglutarate is the source of glutamate. Glutamate is transformed into glutamine, which bacteroids subsequently provide to the host plant. Only partial sections of TCA cycle that function in nitrogen fixation program were specifically up-regulated in *M. huakuii* 7653R.

However, not all reports in the literature are in agreement. For example, up-regulation of TCA-cycle enzymes was recently reported in 28-day-old bacteroids of *R. leguminosarum* [15]. But in *R. etli*, the transcription data suggest that the TCA cycle is largely inactive [41]. A similar down-regulation was observed in *S. meliloti* [49]. Although α-ketoglutarate dehydrogenase mutants of *B. japonicum* have lower rates of nitrogen fixation per plant, they fix N_2_ at the same rate as the wild-type on a per-bacteroid basis [50]. These data suggest that, although α-ketoglutarate dehydrogenase mutants of *B. japonicum* are impaired for normal growth and development, once a bacteroid has formed it does not need a fully functioning TCA cycle. In this present study, we found that all essential steps were tailored to being up-regulated in bacteroids adapt to nitrogen fixation. These results counter that presented in the classical model of N_2_ reduction and suggest that either a bypass of the TCA cycle must operate in bacteroids. For example a α-ketoglutarate decarboxylase step and C4-dicarboxylic acids dehydrogenase step, or that the TCA cycle is non-cyclic, with intermediates leaving at a key branch point.

#### 2.3. Polyhydroxybutyrate (PHB) biosynthesis

In this study, expression of PHB synthesis genes was down-regulated, and those of PHB degradation genes were unchanged. The bacterial carbon storage compound PHB accumulates in rhizobia cells within infection threads [51], [52]. It is possible that intracellular PHB stores may fuel cell division and growth during root infection and invasion as PHB can be used as a carbon and energy source for bacteroid formation. Most species of rhizobia synthesize PHB, but not all accumulate it during symbiosis with legumes. During the formation of bacteroids in indeterminate-type nodules, the PHB granules are broken down. Biochemically, PHB synthesis directly competes with N_2_ fixation for reductant [53]. As acetyl-CoA can either enter the TCA cycle via condensation with oxaloacetate, or form PHB, N_2_-fixing rhizobium bacteroids face a tradeoff between nitrogen fixation and PHB accumulation. The situation in determinate-type nodules is even more complex. Mature bacteroids continue to accumulate large amounts of PHB. One of the reasons might be that N_2_ was fixed as ureides rather than glutamine or asparagine. The other reason might be attributed to the fact that in the determinate-type nodules bacteroids are reversibly differentiated and have the reproductive capacity [3]. Therefore, PHB granules may fuel rhizobial cell division in the determinate-type nodules, but PHB is not essential for the terminally differentiated bacteroids in the indeterminate-type nodules. Trainer and Trevor (2007) demonstrated that PhaP proteins are required for PHB granule accumulation in *S. meliloti* [54]. But *phaP* is absent in the *M. huakuii* 7653R genome, which might be another reason for the lack of PHB accumulation in 7653R indeterminate-type nodules.

#### 2.4. Branched-chain amino acids transport

Rhizobia are capable of utilizing a wide range of compounds from soil and rhizosphere as evident by the large number of transport systems they possess [7], [55]. For example, ATP binding cassette (ABC) transporters are abundant in rhizobia. Amino acid transport in Rhizobia occurs predominantly via two ABC transport systems, L-amino acid permease (Aap) and branched chain amino acid transport system (Bra). Both systems have broad-range specificity for amino acids and are together required for effective symbiotic nitrogen fixation [51]. The Aap, encoded by the *aapJQMP* operon, belongs to the polar amino acid transporter (PAAT) subclass of ABC transport systems [56]. The Bra, encoded by *braC-DEFG*, belongs to the hydrophobic amino acid transporter (HAAT) subclass of ABC transporters [57]. Bacteroids require branched-chain amino acids (LIV) transport, and this cannot be achieved via endogenous glutamate synthesis alone. Thus, although free-living cultures are prototrophic for LIV, bacteroids do become auxotroph [58]. ABC uptake systems are important in symbiosis, but they may only be active prior to bacteroid maturation [59]. Strains of *R. leguminosarum* (with mutations in both *aap* and *bra*) within nodulated peas only fixed N_2_ at around 30% of wild-type rates [51]. In this work, Aap and Bra systems in 7653R, which were homologous to *R. leguminosarum* Aap (AapJQMP) and Bra (BraDEFGC) respectively, were either down-regulated or remained unchanged. It is important to note, that several other *bra* gene clusters organized in one operon in 7653R genome exists, and all are up-regulated (Table 2). This suggests the presence of an alternative transport system for branched-chain amino acids in *M. huakuii* 7653R bacteroids, for which the specific functional mechanism remains to be determined.

**Table 2:** Genes associated with branched-chain amino acid transport in *M. huakuii* 7653R.

GeneID	Gene	Function	Up or Down-Regulation
MCHK_1484	*aapJ*	lysine-arginine-ornithine-binding periplasmic family protein	Unchanged
MCHK_1485	*aapQ*	binding-dependent transport system inner membrane component family protein	Down
MCHK_1486	*aapM*	inner membrane amino-acid ABC transporter permease protein yhdY	Down
MCHK_1487	*aapP*	ABC transporter family protein	Down
MCHK_5281	*braC*	ABC transporter, substrate binding protein	Unchanged
MCHK_5283	*braG*	ABC transporter family protein	Unchanged
MCHK_5284	*braF*	ABC transporter family protein	Unchanged
MCHK_5285	*braE*	branched-chain amino acid transport system/permease component family protein	Unchanged
MCHK_5286	*braD*	branched-chain amino acid transport system/permease component family protein	Unchanged
MCHK_5954	*braG*	ABC transporter family protein	Up
MCHK_5955	*braF*	ABC transporter family protein	Up
MCHK_5956	*braE*	branched-chain amino acid transport system/permease component family protein	Up
MCHK_5957	*braD*	branched-chain amino acid transport system/permease component family protein	Up
MCHK_6267	*braF*	ABC transporter family protein	Up
MCHK_6268	*braD*	branched-chain amino acid transport system/permease component family protein	Up
MCHK_6269	*braE*	branched-chain amino acid transport system/permease component family protein	Up
MCHK_6270	*braC*	branched-chain amino acid transport system substrate-binding protein	Up
MCHK_6271	*braG*	ABC transporter family protein	Up
MCHK_0689	*braE*	branched-chain amino acid transport system/permease component family protein	Up
MCHK_0690	*braD*	branched-chain amino acid transport system/permease component family protein	Up
MCHK_0691	*braG*	ABC transporter family protein	Up
MCHK_0692	*braF*	ABC transporter family protein	Up

#### 2.5. Fatty acid metabolism

Analysis of transcriptome data indicated metabolism of fatty acids was not occurring in the 7653R bacteroids. RNA-Seq and Microarrays detected a variety of *fab* genes participating in fatty acid biosynthesis, yet their expression remained unchanged (Table S9). Fatty acid metabolism plays an important role in bacteroid metabolism as it supplies a variety of precursors, (e.g. components of rhizobial membranes, or lipopolysaccharides and coenzymes required in signal transduction). In the 18 dpi bacteroids, metabolism of fatty acid in *R. etli* is active [45]. In contrast, in the 28 dpi bacteroids, a drastic reduction in lipid biosynthesis was observed in *B. japonicum* [60]. As the host plant does not supply fatty acids; the requirement for lipids is reduced during the later stages of bacteroid nitrogen fixation, due in part to its non-dividing nature. More importantly, fatty acid synthesis requires NADPH as a reducing agent and acetyl-CoA as a precursor. The down-regulation of fatty acid metabolism provides NADPH and acetyl-CoA for use in bacteroid nitrogen fixation during this stage.

### 3. Bacteroid differentiation

#### 3.1. Cell cycle and c-di-GMP

Indeterminate nodules are occupied by a single bacteroid, which has enlarged cells, increased genome amplification, and a loss of membrane integrity and reproductive capacity. In contrast, bacteroids in determinate nodules have no significant morphological differentiation and do maintain their capacity for cell division [3]. The bacteroid development in indeterminate nodules therefore represents a symbiosis-specific bacterial cell cycle event, which is closely connected with either intracellular persistence, or efficient nitrogen fixation. Thus, we sought to better understand how the bacterial cell cycle might be regulated for bacteroid differentiation and nitrogen fixation in indeterminate nodules.

Rhizobia cell cycle regulation has not been studied in great detail, with the exception of that in *S. meliloti* [61]; however, this has been examined in detail in *Caulobacter crescentus* [62]. It is apparent that rhizobia under free-living and symbiotic conditions undergo asymmetrical division as *C. crescentus*; the cell cycle of *C. crescentus* is providing insight for our analysis of cell cycle relevant gene expression in *M. huakuii* 7653R.

The CtrA protein was first characterized for *C. crescentus* [3], in which it is essential for viability and acts as a master regulator of the cell cycle [63]. CtrA controls at least 25% of the genes involved in cell cycle progression, including those required for DNA replication, DNA methylation, cell division, and biogenesis of flagella and pili [64]. The *ctrA* genes of *S. meliloti* [65], *Brucella abortus* [61], *Rhodobacter capsulatus* [66], and *Ruegeria* sp. strain TM1040 [67] have also been studied.

**Figure 5:**
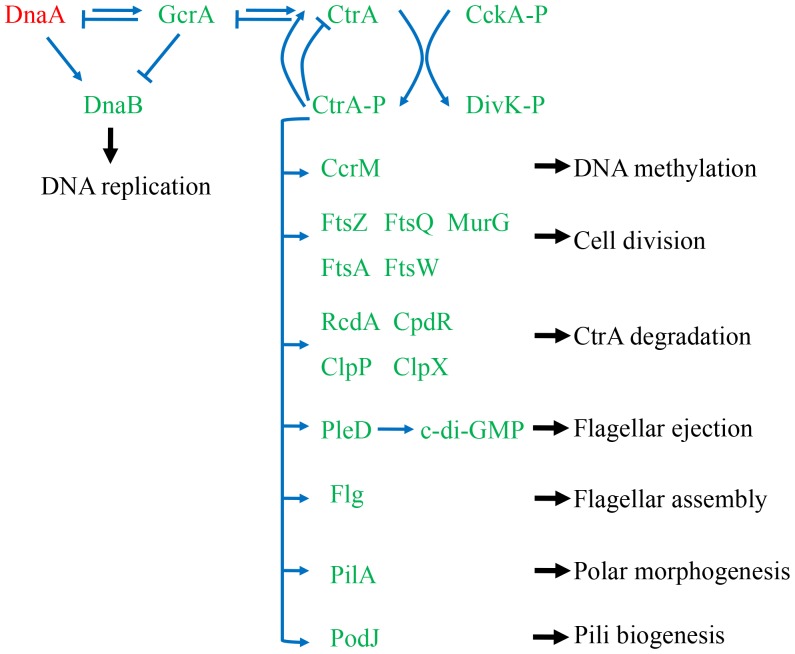
The putative CtrA regulatory network in *M. huakuii* 7653R according to the regulation model of cell cycle in *C. crescentus*. Blue arrows indicate positive effects and blunt blue lines indicate negative effects. Genes up-regulated were shown in red and down-regulated shown in green in *M. huakuii* 7653R bacteroids. Phosphorylated CtrA autoregulated its own transcription and CtrA regulated genes associated with DNA replication, DNA methylation, cell division, flagellar assembly, CtrA degradation, cell division, polar morphogenesis and pili biogenesis.

Based on analysis of RNA-Seq and Microarrays data in this study, in accordance with the center protein CtrA, a significant number of housekeeping genes were down-regulated at the nitrogen fixation stage except *dnaA*, thus we presumed that these genes are associated with the cell cycle (Table S10). The biological functions of these genes were associated with DNA replication, DNA methylation, cell division, flagellar assembly, flagellar ejection, CtrA degradation, polar morphogenesis and pili biogenesis (Figure 5). As DnaA protein activates the initiation of DNA replication in prokaryotes, *dnaA* was up-regulated in bacteroids, demonstrating its agreement with their genome endoreduplication at this stage. The gene expression profiles were congruent with cellular events during bacteroid differentiation, when the cells underwent successive rounds of genome multiplication, without accompanying cell division.

The c-di-GMP relevant genes were down-regulated as well in the bacteroids. The intracellular concentration of c-di-GMP is controlled by diguanylate cyclase (DGC) which contains a GGDEF [68] or GGEEF [69] domain, and hydrolytic enzymes, phosphodiesterases (PDE) containing either a EAL [70] or HD-GYP [71] domain. In the RNA-Seq and Microarrays data of *M. huakuii*, we list genes containing the above conserved protein domains, and their expression (Table S11); most were down-regulated. Interestingly, two genes *mhl3535* and *mhl8229* were up-regulated. This suggests that the function of these two genes might differ from others. Actually, not all EAL or GGDEF domain-containing proteins are enzymatically active as are DGC or PDE. c-di-GMP may be involved in the regulation of bacteria and plant symbiosis [72]. But their function has not been investigated using genetic, or biochemical approaches. It remains to be determined when c-di-GMP plays and important role during nodulation and nitrogen fixation. Thus, the intracellular level and dynamic changes of c-di-GMP in bacteroids should be further studied.

#### 3.2. BacA and TypA

Bacteroids in indeterminate nodules of Inverted Repeat Lacking Clade (IRLC) legumes undergo terminal differentiation caused by Nodule-specific Cysteine-Rich peptides (NCRs) [73], [74]. The BacA protein from *S. meliloti* was the first protein shown to be essential for bacteroid development [75]. It has also been shown to be essential for the symbiosis between *M. huakuii* 7653R and *A. sinicus* [76]. BacA provides a critical protective role for bacteria entering infected plant cells. The essential function of BacA in symbiosis seems limited to indeterminate-nodules-formation host plant, which produce NCRs. However, it remains to be determined whether this occurs via a direct interaction of BacA with the NCR peptides, or whether the peptide transport function of BacA could be involved in maintaining an oxidizing environment within the periplasm.

TypA, also named BipA, belongs to a superfamily of ribosome-binding GTPases within the TRAFAC class (translation factors) of GTPases [77], [78]. TypA has been suggested to be involved in the regulation of virulence and stress responses in pathogenic *E. coli* [79], *Salmonella enterica* Serovar Typhimurium [80] and *Pseudomonas aeruginosa* [81], and stress responses in non-pathogenic *S. meliloti* [82] and *Bacillus subtilis*.[83].

The establishment of the Rhizobium-legume symbiosis is similar to pathogenesis in several respects. In both cases, bacteria have to penetrate the plant tissues, avoid the host defenses, and adapt to the physiological conditions found in the new environment [84], [85]. During nodule invasion and bacteroid differentiation, rhizobia are exposed to new physiological conditions (pH, osmotic concentration, oxidative stress, defense responses and NCR peptides). TypA was required for survival of *S. meliloti* under certain stress conditions, such as growth at low temperature or low pH and in the presence of sodium dodecyl sulfate (SDS). It is noteworthy that TypA is involved in bacteroid development and is indispensable for symbiosis depending on the host plant type (indeterminate-nodules-formation host plant) [82]. This demonstrates that TypA and BacA have highly similarity in symbiotic phenotypes. It is promoted us to speculate the possible functional associations between TypA and BacA.

In a parallel study, we found that *M. huakuii* 7653R TypA protein interacts with BacA in a bacterial two-hybrid system. In addition, a *typA* knockout mutant exhibited abnormal development of bacteroids in 7653R (unshown) with highly similarity to the symbiotic phenotypes of the *bacA* mutant. Consistent with these data, the gene expressions of *bacA* and *typA* are both up-regulated in bacteroids. These results suggest that TypA, as a regulatory factor, might regulate the activity of BacA and therefore jointly mediate bacteroid differentiation.

### 4. Mapping gene expression dataset into 7653R PPI network

The *M. huakuii* 7653R genome has been recently sequenced (Accession Nos. CP006581-CP006583, http://www.ncbi.nlm.nih.gov/genome/11322). It encodes around 7000 proteins; nearly 40% of them are of unknown function, although some are defined “belonging to a superfamily”. High-throughput experimental data can provide valuable information for linking unknown genes with specific biological roles; and is thus widely used to predict gene function. For example, much protein-protein interaction (PPI) data has been acquired for several microorganisms [28], [86]–[88]. Global viewing of PPI is also a powerful tool for assigning biological role to large numbers of proteins predicted by whole-genome sequencing. Identification of interactions between characterized and uncharacterized proteins infers functional relatedness, thus integrative analysis of datasets may reveal function of uncharacterized genes. Accordingly, we constructed PPI networks for *M. huakuii* 7653R based on the two published PPI reference networks of *M. huakuii* bv *loti* MAFF303099 [28] and *E. coli* [29] published. The former MAFF303099 strain belongs to the same genus and its genome is highly similar to *M. huakuii* 7653R. The inferred PPI network of *M. huakuii* 7653R has 2607 nodes and 10307 edges (Table S1). We integrated gene expression data and protein interaction to identify inter-connected, differentially-expressed components, which may represent important pathways or protein complexes that are altered in bacteroids of 7653R.

### 4.1. Nitrogen fixation

**Figure 6:**
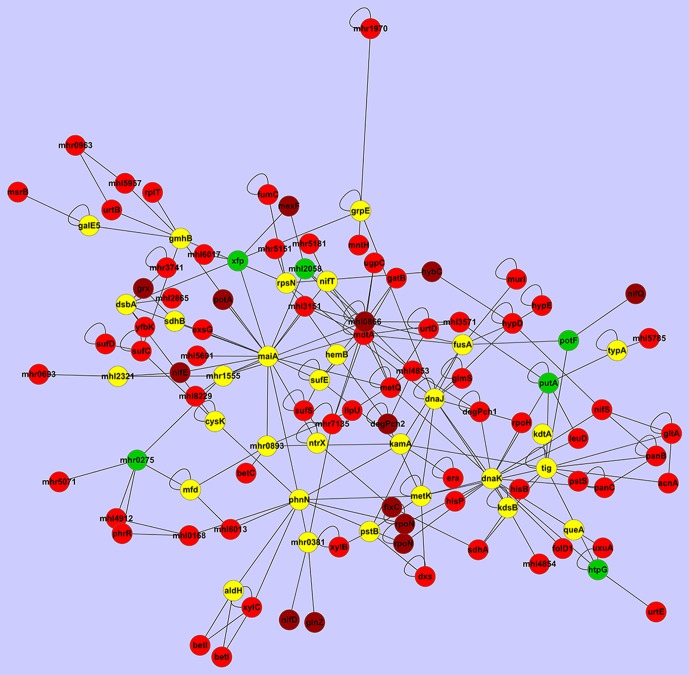
An inter-connected, differentially-expressed PPI subnetwork in bacteroids altered for nitrogen fixation. The genes with differential expression fold change greater than 5 were shown in red; those greater than 20 were shown in dark red; those down-regulated were shown in green and others were shown in yellow.

By selecting those genes with a 5-fold change in expression and mapping the data into the PPI network of 7653R, an interesting subnetwork was identified (Figure 6, Table 3, see Methods). The subnetwork had 113 nodes and 214 edges, including 78 genes with 5-fold change in expression. Enriched GO term analysis found that the most significant GO term was that over-represented were those involved with nitrogen fixation (Figure S4). These results indicate that the constructed PPI network of 7653R and the transcriptomes data identified in this study were reliable.

**Table 3:** The set of genes in the *M. huakuii* 7653R subnetwork of nitrogen fixation.

ID	Name	Function	Fold change
MCHK_8175	*nifD*	nitrogenase molybdenum-iron protein alpha chain	228.1625
MCHK_8172	*nifE*	nitrogenase MoFe cofactor biosynthesis protein NifE	272.4621
MCHK_8188	*nifQ*	nifQ family protein	36.087
MCHK_1835	*nifS*	aminotransferase class-V family protein	11.1585
MCHK_8164	*nifT*	putative nitrogen fixation protein FixU	2.7712
MCHK_8219	*fixC*	FAD dependent oxidoreductase family protein	153.2337
MCHK_2178	*ntrX*	response regulator	0.9891
MCHK_5476	*glnZ*	PII-like protein Pz	43.3614
MCHK_2333	*gltA*	citrate (Si)-synthase	6.0617
MCHK_8190	*rpoN*	sigma-54 factor, core binding domain protein	28.7755
MCHK_7137	*rpoN*	RNA polymerase sigma-54 factor	40.125
MCHK_5199	*rpoH*	RNA polymerase sigma-32 factor	5.7607
MCHK_2096	*rpsN*	30S ribosomal protein S14	0.5787
MCHK_1837	*sufC*	feS assembly ATPase SufC	6.589
MCHK_1838	*sufD*	feS assembly protein SufD	7.4377
MCHK_1111	*sufE*	fe-S metabolism associated domain protein	0.5652
MCHK_1839	*sufS*	cysteine desulfurase, SufS subfamily protein	9.8869
MCHK_2452	*urtB*	urea ABC transporter, permease protein UrtB	16.8354
MCHK_2450	*urtD*	urea ABC transporter, ATP-binding protein UrtD	9.3897
MCHK_2449	*urtE*	urea ABC transporter, ATP-binding protein UrtE	5.1336
MCHK_8359	*hybC*	nickel-dependent hydrogenase family protein	52.028
MCHK_8347	*hypD*	hydrogenase expression/formation protein HypD	5.6356
MCHK_8345	*hypE*	hydrogenase expression/formation protein HypE	5.6193
MCHK_5539	*acnA*	aconitate hydratase 1	9.0088
MCHK_0267	*aldH*	aldehyde dehydrogenase family protein	0
MCHK_1080	*betC*	choline-sulfatase	5.9333
MCHK_1499	*betI*	transcriptional repressor BetI	5.379
MCHK_1081	*betI*	transcriptional repressor BetI	7.0643
MCHK_5793	*cysK*	cysteine synthase A	0
MCHK_6087	*degPch1*	protease Do family protein	12.494
MCHK_1148	*degPch2*	protease Do family protein	22.5974
MCHK_5827	*dnaJ*	chaperone protein DnaJ	0.6215
MCHK_5828	*dnaK*	chaperone protein DnaK	1.7466
MCHK_1003	*dsbA*	DSBA-like thioredoxin domain protein	0.6718
MCHK_0964	*dxs*	1-deoxy-D-xylulose-5-phosphate synthase	5.6548
MCHK_1205	*era*	GTP-binding protein Era	6.0056
MCHK_3376	*exsG*	putative two-component sensor histidine kinase protein	6.334
MCHK_8419	*folD1*	tetrahydrofolate dehydrogenase/cyclohydrolase, catalytic domain protein	8.8978
MCHK_1496	*fumC*	fumarate hydratase, class II	7.3542
MCHK_2080	*fusA*	translation elongation factor G	0.9551
MCHK_0391	*galE5*	short chain dehydrogenase family protein	0.9348
MCHK_4109	*gatB*	yqey-like family protein	14.0403
MCHK_7093	*glmS*	glutamine-fructose-6-phosphate transaminase	8.8581
MCHK_4716	*grpE*	grpE family protein	1.1287
MCHK_5101	*grx*	redoxin family protein	34.8731
MCHK_1705	*hemB*	delta-aminolevulinic acid dehydratase	1.0837
MCHK_6079	*hisB*	imidazoleglycerol-phosphate dehydratase family protein	6.858
MCHK_5153	*hisP*	ABC transporter family protein	5.6978
MCHK_3552	*htpG*	histidine kinase-, DNA gyrase B-, and HSP90-like ATPase family protein	0.3735
MCHK_3523	*kamA*	kamA family protein	1.2954
MCHK_6499	*kdsB*	3-deoxy-D-manno-octulosonate cytidylyltransferase	0.8333
MCHK_1589	*kdtA*	3-Deoxy-D-manno-octulosonic-acid transferase family protein	5.6107
MCHK_5594	*leuD*	3-isopropylmalate dehydratase, small subunit	5.2354
MCHK_3575	*llpU*	short chain dehydrogenase family protein	5.5047
MCHK_2788	*maiA*	maleylacetoacetate isomerase	1.0738
MCHK_4398	*mdtA*	efflux transporter, RND family, MFP subunit	5.1515
MCHK_6536	*metK*	methionine adenosyltransferase	0.9219
MCHK_5860	*metQ*	conserved hypothetical protein	5.4162
MCHK_0867	*mexF*	RND transporter, hydrophobe/amphiphile efflux-1 family protein	58.6485
MCHK_2510	*mfd*	transcription-repair coupling factor	1.5562
MCHK_4118	*mntH*	metal ion transporter, metal ion family protein	14.1307
MCHK_5902	*msrB*	methionine-R-sulfoxide reductase	10.4404
MCHK_1856	*murI*	glutamate racemase	7.7059
MCHK_1985	*panB*	3-methyl-2-oxobutanoate hydroxymethyltransferase	6.6055
MCHK_1984	*panC*	pantoate—beta-alanine ligase	6.8577
MCHK_6052	*phnN*	phosphonate metabolism protein/1,5-bisphosphokinase (PRPP-forming) PhnN	2.4099
MCHK_6535	*phrR*	helix-turn-helix family protein	10.2277
MCHK_1763	*potA*	ABC transporter family protein	23.1647
MCHK_0211	*potF*	bacterial extracellular solute-binding family protein	0.3783
MCHK_5082	*pstB*	phosphate ABC transporter, ATP-binding protein	2.405
MCHK_5085	*pstS*	phosphate ABC transporter, phosphate-binding protein PstS	6.2484
MCHK_2812	*putA*	delta-1-pyrroline-5-carboxylate dehydrogenase	0.2091
MCHK_2418	*queA*	S-adenosylmethionine:tRNA ribosyltransferase-isomerase	0
MCHK_6121	*rplT*	ribosomal protein L20	5.046
MCHK_5489	*sdhA*	succinate dehydrogenase, flavoprotein subunit	5.6783
MCHK_6358	*sdhB*	universal stress family protein	1.4018
MCHK_2704	*tig*	trigger factor	1.6301
MCHK_5374	*typA*	GTP-binding protein TypA/BipA	3.7535
MCHK_3307	*ugpC*	ABC transporter family protein	6.1079
MCHK_0451	*uxuA*	mannonate dehydratase	16.059
MCHK_3900	*xfp*	D-xylulose 5-phosphate/D-fructose 6-phosphate phosphoketolase family protein	0.2442
MCHK_8196	*xylB*	aryl-alcohol dehydrogenase	5.2102
MCHK_8197	*xylC*	benzaldehyde dehydrogenase NAD+	19.882
MCHK_2864	*yfbK*	von Willebrand factor type A domain protein	13.9193
MCHK_0866	*mhl0866*	efflux transporter, RND family, MFP subunit	40.6938
MCHK_2865	*mhl2865*	RNA polymerase sigma factor, sigma-70 family protein	15.6472
MCHK_4854	*mhl4854*	luciferase-like monooxygenase family protein	7.7335
MCHK_4912	*mhl4912*	bacterial regulatory helix-turn-helix, lysR family protein	12.7467
MCHK_5957	*mhl5957*	branched-chain amino acid transport system/permease component family protein	6.4752
MCHK_8229	*mhl8229*	sensory box protein	8.4814
MCHK_0275	*mhr0275*	tetratricopeptide repeat family protein	0.4469
MCHK_0381	*mhr0381*	chain length determinant family protein	1.1847
MCHK_0693	*mhr0693*	bacterial regulatory s, gntR family protein	10.9707
MCHK_1555	*mhr1555*	marR family protein	0.8471
MCHK_5071	*mhr5071*	HWE histidine kinase family protein	5.2004
MCHK_5151	*mhr5151*	amino ABC transporter, permease, 3-TM region, His/Glu/Gln/Arg/opine family domain protein	7.8098
MCHK_4160^a^	*gmhB*	mll2559 protein	1.3228
MCHK_0168^a^	*mhl0168*	mll6478 protein	5.9853
MCHK_6013^a^	*mhl6013*	mll4959 protein	6.0062
MCHK_0893^a^	*mhr0893*	putative uncharacterized protein	1.5161
MCHK_2058^a^	*mhl2058*	conserved hypothetical protein	0.4313
MCHK_2321^a^	*mhl2321*	putative esterase/lipase/thioesterase	0.8216
MCHK_5691^a^	*mhl5691*	putative uncharacterized domain protein	8.5342
MCHK_3151^a^	*mhl3151*	ykud domain protein	8.1921
MCHK_1970^a^	*mhr1970*	bacterial transferase hexapeptide family protein	8.2936
MCHK_3571^a^	*mhl3571*	ABC transporter family protein	12.0058
MCHK_4853^a^	*mhl4853*	flavin reductase like domain protein	5.2647
MCHK_5785^a^	*mhl5785*	FMN-binding domain protein	6.9947
MCHK_6017^a^	*mhl6017*	bacterial regulatory s, lacI family protein	5.7227
MCHK_0963^a^	*mhr0963*	pirin C-terminal cupin domain protein	5.4469
MCHK_3741^a^	*mhr3741*	periplasmic binding s and sugar binding domain of the LacI family protein	6.9341
MCHK_5181^a^	*mhr5181*	bacterial regulatory s, tetR family protein	7.5806
MCHK_7135^a^	*mhr7135*	putative peroxiredoxin sll1621	14.553

a : The unknown function genes.

An overview analysis on the biological function of gene set (Table 3), indicated that genes in the subnetwork reflected the main features of symbiotic nitrogen fixation. (e.g. highly expressed genes such as *nif*, *fix*, *suf*, sigma factor σ^54^, ammonium assimilation associated, amino acid transport, etc.). Nif and Fix are important components of the symbiotic nitrogen fixation machinery. The *nif* gene encodes dinitrogenase, nitrogenase reductase, and iron-molybdenum cofactor synthesis proteins, whereas the *fix* gene encodes electron transfer relevant ferredoxins or flavoproteins. The *Suf* gene is involved in iron-sulfur (Fe-S) cluster assembly. SufS is cysteine desulfurase, similar to NifS, and supplies sulfur for Fe-S centers. The SufBCD complex activates the cysteine desulfurase activity of SufS in conjunction with the SufE that is a sulfur acceptor protein. The high expression of these *suf* genes suggests that bacteroids require a large amount of Fe-S clusters and that Fe-S clusters provided by SufS proteins may be used to sustain nitrogen fixation as well as the NifS protein. Lastly, although it has been previously reported that expression levels of genes encoding the PII-like protein of *R. etli* [89] and *S. meliloti* [90] are lower in bacteroids than in free-living cells, our results suggest otherwise, as *MCHK_5476* (PII-like protein Pz) was highly expressed in 7653R bacteroids. This suggests that transcriptional regulation of nitrogen sensing and assimilation in *M. huakuii* 7653R bacteroids is different from that of *R. etli* and *S. meliloti*. In addition, several amino acid transport system associated proteins were also members of the identified subnetwork, reflecting the importance of amino acids, particularly branched-chain amino acids that are exchanged between bacteroids and host cells (refer to the section 3.2.4). It is interesting to note the importance of *rpoH* (RNA polymerase sigma-32 factor) in the nitrogen fixation subnetwork. The transcriptional regulation of nitrogen fixation associated genes is generally mediated through RpoN, a RNA polymerase sigma-54 factor. In *S. meliloti*, RpoH1 is required for nitrogen-fixing symbiosis with alfalfa, the *rpoH1* mutant exhibits a nitrogen fixation defect (Fix^−^) and the *rpoH1* is expressed within a symbiotic nodule [91]. Similarly in 7653R, *MCHK_5199* (encoding RNA polymerase sigma-32 factor) is up-regulated, which supports RpoH as having an essential role in the PPI complex of nitrogen fixation. Some hub genes which can be simultaneously targeted by a comparatively large number of genes are related to the resistance/nodulation/cell division (RND) family, such as *MCHK_0866* and *MCHK_4398*. Transport systems of the RND family are the major cause of antibiotic resistance in clinically relevant Gram-negative bacteria [92]. Lindemann, et al reported that a mutant lacking this predicted RND transport system (designated BdeAB), confers antibiotic resistance and is required for an efficient symbiosis; specifically within soybean [93]. This suggests that the RND family might be involved in nitrogen fixation. Earlier studies have demonstrated that two genes encoding a putative multidrug efflux pump of the RND/MFP family are co-transcribed with an *rpoH* gene in *B. japonicum* [94]. Our results were similar in that RpoH and RND family proteins were in the subnetwork of nitrogen fixation. Lastly, nearly 15% of the genes in the subnetwork were of “unknown function”; are ascribed to novel candidates essential to nitrogen fixation; and therefore certainly worthy of further examination.

#### 4.2. CtrA-centered cell cycle

**Figure 7:**
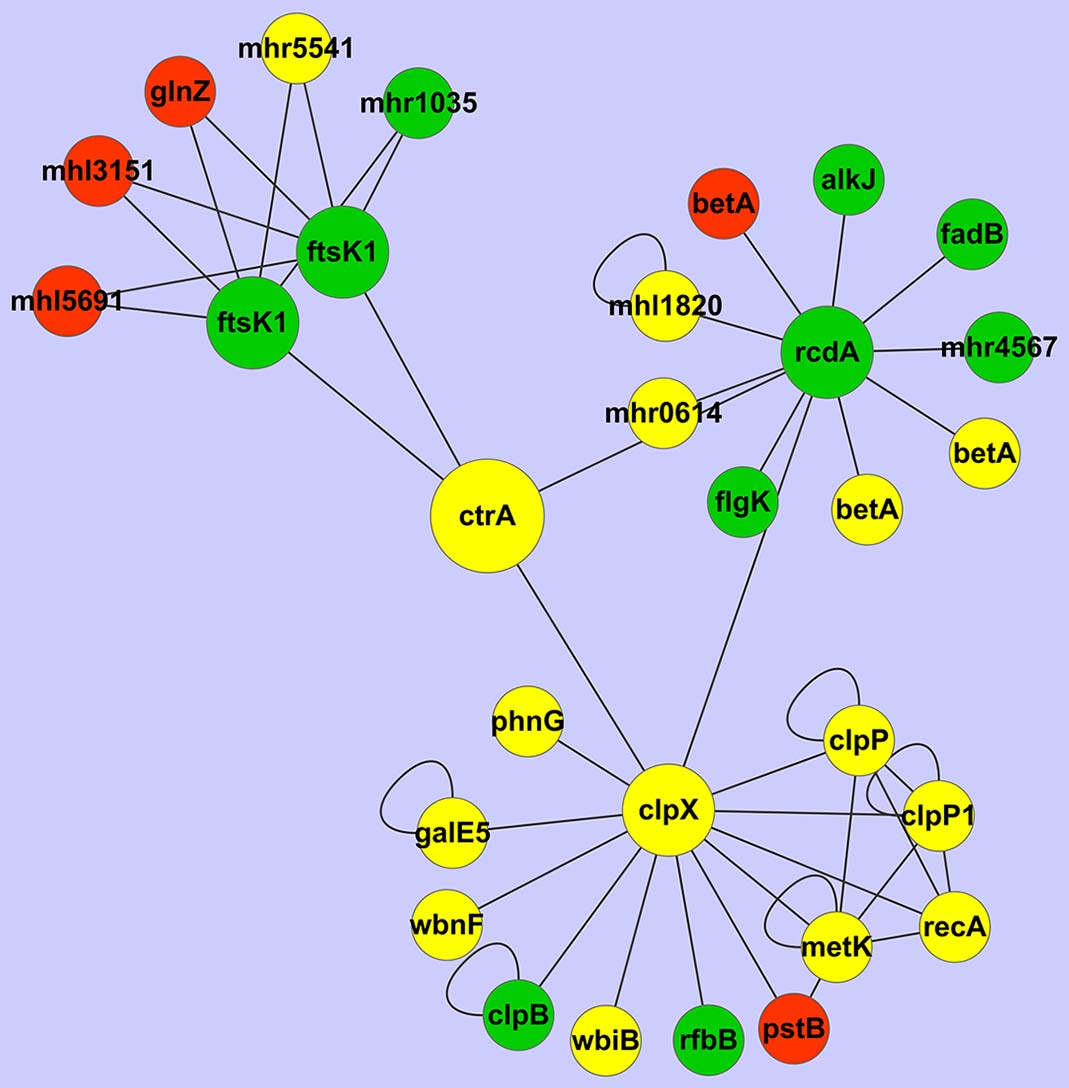
The subnetwork of CtrA and its interacting proteins in *M. huakuii* 7653R. The genes up-regulated were shown in red, those down-regulated were shown in green and those of no change were shown in yellow.

In order to identify the subnetwork mediated by CtrA connected with the cell cycle and bacteroid differentiation, we dissected the 7653R PPI network by using CtrA as a specific target. Then we analyzed this CtrA subnetwork which consists of 30 genes (Table 4, Figure 7). Based on the analysis of RNA-Seq and Microarrays data we found that expression levels for most of the genes in the subnetwork were down-regulated or unchanged. It was found that four genes directly interacting with CtrA, which regulated cell cycle progression [95]–[98]. In particular, six genes (*MCHK_0391*, *MCHK_3151*, *MCHK_5082*, *MCHK_5476*, *MCHK_5691* and *MCHK_6536*) present in the CtrA-centered subnetwork (Table 4), existed in the “nitrogen fixation” subnetwork (Table 3) as well. Among these genes, the PII-like protein Pz was a nitrogen sensing protein [99]; function of the remaining genes is unknown. Therefore, the CtrA subnetwork might be related to nitrogen fixation through a yet to be determined “cross-talk” mechanism. Several phosphate ABC transporters, responsible for phosphate uptake, were also found. This suggests the involvement of some level of coordination to meet the higher phosphate demand that occurs during bacteroid nitrogen fixation. There were also several glucose-methanol-choline (GMC) oxidoreductase family proteins in the CtrA-centered subnetwork. This is perhaps not surprising, as oxidation-reduction reactions are a fundamental reaction occurring in all organisms. The family of glucose–GMC oxidoreductases is represented by a wide variety of enzymes present in both prokaryotic and eukaryotic organisms. The previously unidentified GMC oxidoreductase gene was present in the subnetwork of 7653R; it is plausible that this gene is involved in the cell cycle. This subnetwork provided novel insights into the CtrA-centralized network in *M. huakuii* 7653R relevant to cell cycle, cell differentiation, and nitrogen sensing in bacteroids.

**Table 4:** The subnetwork of CtrA with interacting proteins in *M. huakuii* 7653R.

ID	Name	Function	Up or Down-Regulation
MCHK_1078	*betA*	choline dehydrogenase	Up
MCHK_5082^a^	*pstB*	phosphate ABC transporter, ATP-binding protein	Up
MCHK_5476^a^	*glnZ*	PII-like protein Pz	Up
MCHK_5691^a^	*mhl5691*	putative uncharacterized domain protein	Up
MCHK_3151^a^	*mhl3151*	ykud domain protein	Up
MCHK_4201	*betA*	GMC oxidoreductase family protein	Unchanged
MCHK_0414	*betA*	GMC oxidoreductase family protein	Unchanged
MCHK_0614	*mhr0614*	GMC oxidoreductase family protein	Unchanged
MCHK_1015	*wbnF*	short chain dehydrogenase family protein	Unchanged
MCHK_0391^a^	*galE5*	short chain dehydrogenase family protein	Unchanged
MCHK_1025	*wbiB*	NAD dependent epimerase/dehydratase family protein	Unchanged
MCHK_1847	*recA*	protein RecA	Unchanged
MCHK_2439	*clpP1*	clp protease family protein	Unchanged
MCHK_4371	*ctrA*	response regulator	Unchanged
MCHK_6536^a^	*metK*	methionine adenosyltransferase	Unchanged
MCHK_1820	*mhl1820*	putative oxidoreductase	Unchanged
MCHK_5541	*mhr5541*	putative uncharacterized domain protein	Unchanged
MCHK_5341^b^	*ftsK1*	ftsK/SpoIIIE family protein	Down
MCHK_5471^b^	*ftsK1*	ftsK/SpoIIIE family protein	Down
MCHK_4567	*mhr4567*	GMC oxidoreductase family protein	Down
MCHK_4720	*alkJ*	GMC oxidoreductase family protein	Down
MCHK_1789	*clpP*	ATP-dependent Clp protease, proteolytic subunit ClpP	Down
MCHK_1790^b^	*clpX*	ATP-dependent Clp protease, ATP-binding subunit ClpX	Down
MCHK_6106	*fadB*	UDP-glucose/GDP-mannose dehydrogenase family, NAD binding domain protein	Down
MCHK_1018	*rfbB*	dTDP-glucose 4,6-dehydratase	Down
MCHK_4463	*flgK*	flagellar hook-associated protein FlgK	Down
MCHK_4772	*phnG*	phosphonate C-P lyase system protein PhnG	Down
MCHK_4842	*clpB*	ATP-dependent chaperone ClpB	Down
MCHK_5239^b^	*rcdA*	regulator of CtrA degradation	Down
MCHK_1035	*mhr1035*	short chain dehydrogenase family protein	Down

a The protein also in the nitrogen fixation subnetwork.

b The protein interacts directly with CtrA.

## Conclusion

In the past decade, several genome-wide transcriptome studies have described differential gene expression in bacteroids. These studies identified many genes involved in nitrogen-fixing symbiosis. However, the RNA-Seq method used in this study (in combination with Microarrays) examines a larger range of differential gene expression levels. In addition, we developed an improved method for the isolation and purification of bacteroid RNA for use in subsequent transcriptome analysis of this, and other Rhizobium-legume symbiosis systems. To examine the regulatory mechanisms involved in bacteroid differentiation, we focused our analysis on the putative PPI subnetwork, and the molecules participating in bacteroid differentiation. We found that CtrA/c-di-GMP may be involved in regulation of the cell cycle, bacteroid development, and nitrogen fixation. These data also suggest that *bacA/typA* might coordinately mediate bacteroid differentiation, and adaption to intracellular environments of infected host cells. In order to further understand how the genes determine the progression to bacteroid differentiation and nitrogen fixation, we expanded our analyses to include interaction of genes, rather than a single, or at best several gene functions. We integrated gene expression data and protein interaction data of 7653R to examine interaction between differentially expressed genes. Functional enrichment analysis revealed that biological processes are altered in nodule bacteroids compared with free-living cells. A few hubs include critical proteins or transcription factors, which alter expression level and therefore have a significant role in bacteroid differentiation and nitrogen fixation. Hence, the results described in this study provide a global biological landscape and novel insight that advances our understanding of bacteroid differentiation and nitrogen fixation in indeterminate-type root nodules. It also provides a valuable dataset for further functional confirmation, and use as a reference for investigation of other Rhizobium-legume symbiosis systems.

## Supporting Information

Figure S1 The workflow of constructing PPI networks for *M. huakuii* 7653R. A: the schematic of the homologous protein-protein interactions network through the concepts of orthologous conservation (or interologs); B: the schematic illustration of the search for *M. huakuii* 7653R protein–protein interactions.
(TIFF)

Figure S2 The distribution of differentially expressed genes (bacteroids vs. free-living cells) which account for 80% of total expression amounts in COG functional categories. All genes were assigned to 21 COG functional categories using RPS-BLAST (uncategorized genes not shown). Bars represent the numbers of corresponding genes in free-living cells (green) and bacteroids (blue). The number in each bar represents its percentage (%).
(TIFF)

Figure S3 Schematic view of expressions of known genes associated with pathways of important biological function. An overview of the differentially expressed genes in bacteroids compared with free-living cells. The up-regulated genes are shown in red; the down-regulated genes are shown in green; the unchanged genes are shown in black. Genes were grouped according to their biological function or process involved; membrane-associated proteins were positioned over the inner and outer membranes.
(TIFF)

Figure S4 Up-regulated GO terms associated with bacteroids identified by BiNGO plugin. The enriched GO term of the subnetwork was nitrogen fixation.
(TIFF)

Table S1 The PPI network of *M. huakuii* 7653R inferred from the two published PPI reference networks of *M. huakuii* bv *loti* MAFF303099 and *E. coli*.
(XLSX)

Table S2 The top 10 of highly-expressed genes in *M. huakuii* 7653R bacteroids revealed by RNA-Seq. ^a^ The percentage represents the proportion of the gene transcriptional amounts to the total transcriptional amounts.
(DOC)

Table S3 Top 25 up-regulated genes in *M. huakuii* 7653R bacteroids revealed by RNA-Seq and Microarrays.
(DOC)

Table S4 Top 25 down-regulated genes in *M. huakuii* 7653R bacteroids revealed by RNA-Seq and Microarrays.
(DOC)

Table S5 The significantly up-regulated pathways based on GSEA in *M. huakuii* 7653R free-living cells.
(XLSX)

Table S6 The significantly up-regulated pathways based on GSEA in *M. huakuii* 7653R.
(XLSX)

Table S7 Enriched KEGG pathways of differential expression genes in *M. huakuii* 7653R free-living cells.
(XLSX)

Table S8 **Enriched KEGG pathways of differential expression genes in *M. huakuii* 7653R.**
(XLSX)

Table S9 *fab* genes participating in fatty acid biosynthesis in *M. huakuii* 7653R.
(XLSX)

Table S10 Genes associated with cell cycle and putatively regulated by CtrA in *M. huakuii* 7653R.
(XLSX)

Table S11 Genes associated with c-di-GMP synthesis and degradation in *M. huakuii* 7653R.
(XLSX)
