# Supplementary figures and images for: RNA-Seq and Microarrays Analyses Reveal Global Differential Transcriptomes of Mesorhizobium huakuii 7653R between Bacteroids and Free-Living Cells
Source: PLoS One. 2014 Apr 2;9(4):e93626. doi: 10.1371/journal.pone.0093626 (PMC3973600; doi:10.1371/journal.pone.0093626)

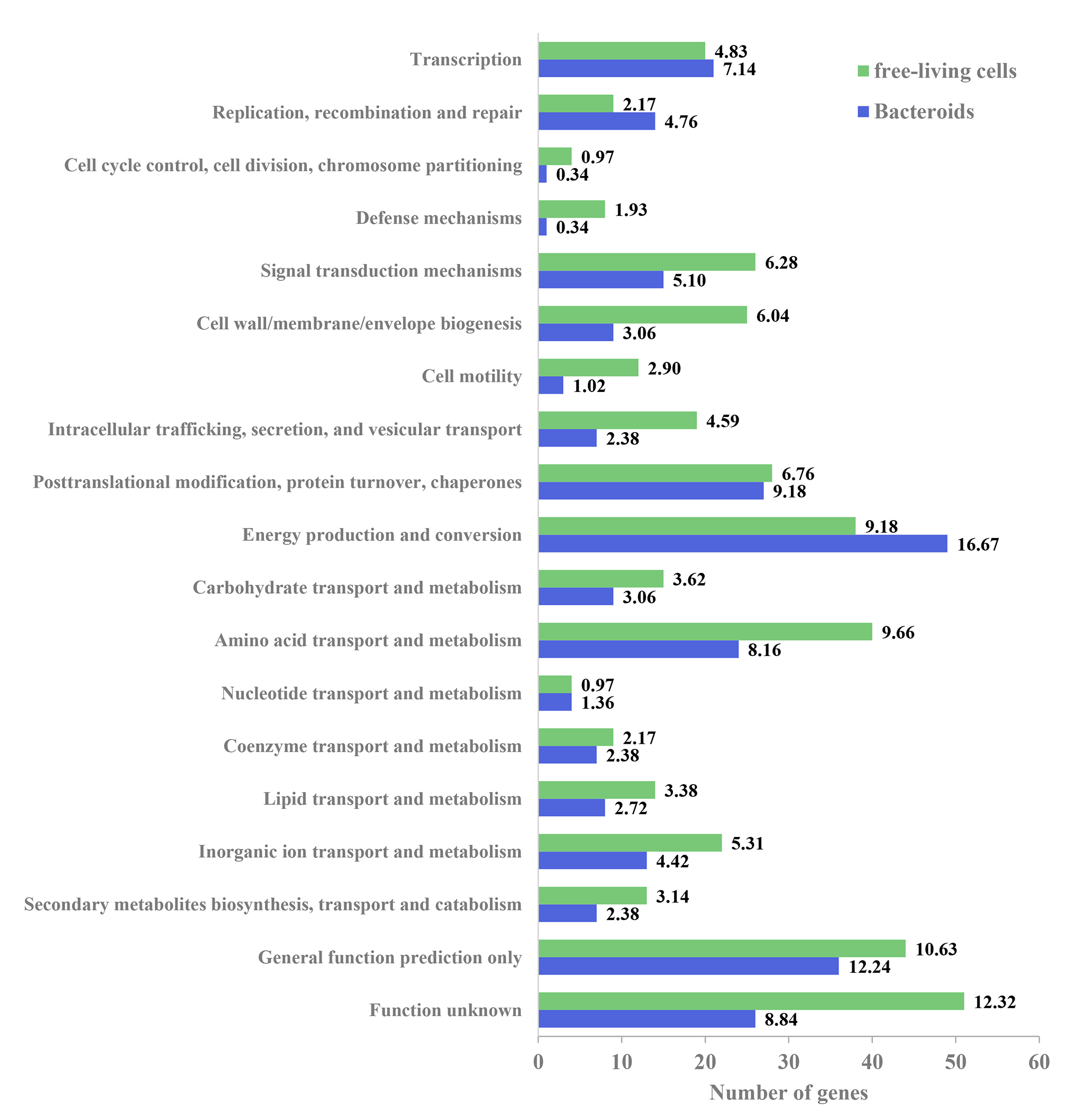

Supplement: Figure S2 — The distribution of differentially expressed genes (bacteroids vs. free-living cells) which account for 80% of total expression amounts in COG functional categories. All genes were assigned to 21 COG functional categories using RPS-BLAST (uncategorized genes not shown). Bars represent the numbers of corresponding genes in free-living cells (green) and bacteroids (blue). The number in each bar represents its percentage (%). (TIFF) [file pone.0093626.s002.tiff]

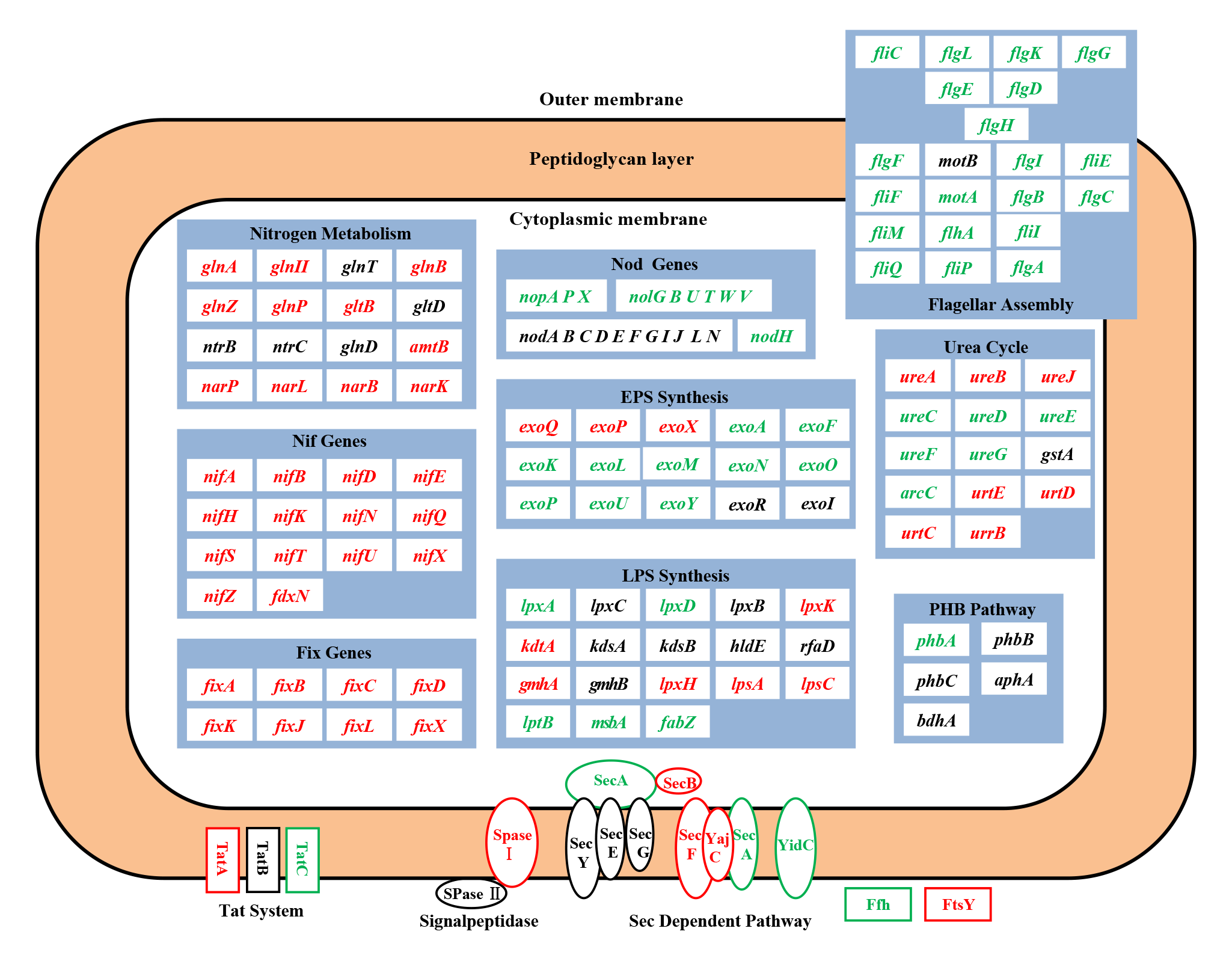

Supplement: Figure S3 — Schematic view of expressions of known genes associated with pathways of important biological function. An overview of the differentially expressed genes in bacteroids compared with free-living cells. The up-regulated genes are shown in red; the down-regulated genes are shown in green; the unchanged genes are shown in black. Genes were grouped according to their biological function or process involved; membrane-associated proteins were positioned over the inner and outer membranes. (TIFF) [file pone.0093626.s003.tiff]

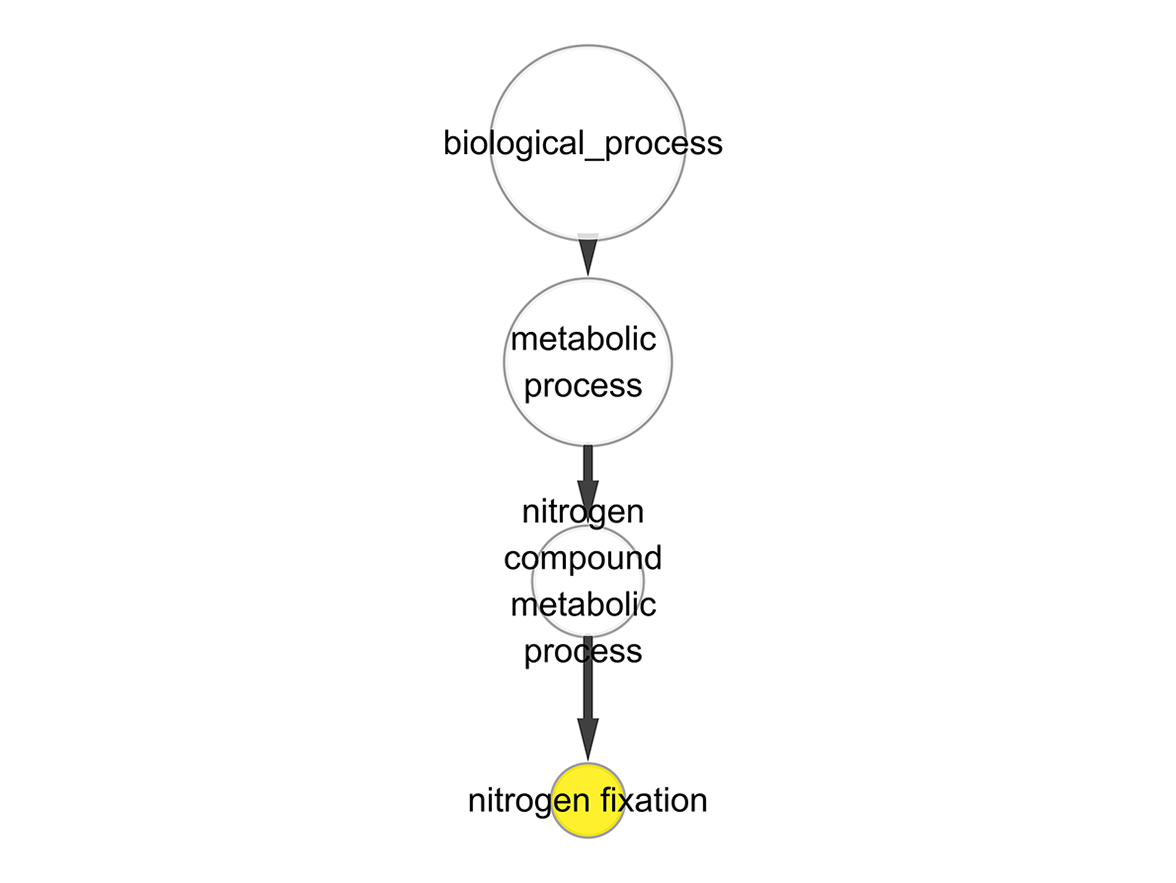

Supplement: Figure S4 — Up-regulated GO terms associated with bacteroids identified by BiNGO plugin. The enriched GO term of the subnetwork was nitrogen fixation. (TIFF) [file pone.0093626.s004.tiff]
