# Supplementary material for: RNA-Seq and Microarrays Analyses Reveal Global Differential Transcriptomes of Mesorhizobium huakuii 7653R between Bacteroids and Free-Living Cells
Source: PLoS One. 2014 Apr 2;9(4):e93626. doi: 10.1371/journal.pone.0093626 (PMC3973600; doi:10.1371/journal.pone.0093626)
Supplement: Table S2 — The top 10 of highly-expressed genes in M. huakuii 7653R bacteroids revealed by RNA-Seq. a The percentage represents the proportion of the gene transcriptional amounts to the total transcriptional amounts. (DOC) [file pone.0093626.s006.doc]

**Table S2. The top 10 of highly-expressed genes in *M. huakuii* 7653R bacteroids revealed by RNA-Seq**

| GeneID | Gene | Function | Percentage^a^ in bacteroids | Percentage^a^ in free-living cells |
| --- | --- | --- | --- | --- |
| MCHK_8175 | *nifD* | nitrogenase molybdenum-iron protein alpha chain | 7.33% | 0.00% |
| MCHK_8176 | *nifH* | nitrogenase iron protein | 5.47% | 0.00% |
| MCHK_8169 | *nifX* | nitrogen fixation protein NifX | 5.16% | 0.00% |
| MCHK_1372 | *mhr1372* | conserved hypothetical protein | 3.37% | 0.17% |
| MCHK_8174 | *nifK* | nitrogenase molybdenum-iron protein beta chain | 2.63% | 0.00% |
| MCHK_8217 | *fixA* | electron transfer flavodomain protein | 2.01% | 0.00% |
| MCHK_7192 | *mhr7192* | hypothetical protein | 1.62% | 0.00% |
| MCHK_7139 | *mhl7139* | hypothetical protein | 1.59% | 0.00% |
| MCHK_8172 | *nifE* | nitrogenase MoFe cofactor biosynthesis protein NifE | 1.52% | 0.00% |
| MCHK_7131 | *yoaF* | conserved hypothetical protein | 1.47% | 0.00% |

^a^ The percentage represents the proportion of the gene transcriptional amounts to the total transcriptional amounts.
