# Supplementary material for: RNA-Seq and Microarrays Analyses Reveal Global Differential Transcriptomes of Mesorhizobium huakuii 7653R between Bacteroids and Free-Living Cells
Source: PLoS One. 2014 Apr 2;9(4):e93626. doi: 10.1371/journal.pone.0093626 (PMC3973600; doi:10.1371/journal.pone.0093626)
Supplement: Table S3 — Top 25 up-regulated genes in M. huakuii 7653R bacteroids revealed by RNA-Seq and Microarrays. (DOC) [file pone.0093626.s007.doc]

**Table S3. Top 25 up-regulated genes in *M. huakuii* 7653R bacteroids revealed by RNA-Seq and Microarrays**

| **RNA-Seq** | | | | Microarrays | | | |
| --- | --- | --- | --- | --- | --- | --- | --- |
| GeneID | Gene | log2 Ratio | P-value | GeneID | Gene | log2 Ratio | Q-value (%) |
| MCHK_5475 | *mhr5475* | 17.52 | 6.66E-16 | MCHK_8176 | *nifH* | 8.16 | 0 |
| MCHK_3354 | *mhl3354* | 13.89 | 1.02E-07 | MCHK_8172 | *nifE* | 8.09 | 0 |
| MCHK_8176 | *nifH* | 13.35 | 1.56E-05 | MCHK_8174 | *nifK* | 7.94 | 0 |
| MCHK_7064 | *mhr7064* | 13.10 | 2.75E-05 | MCHK_8175 | *nifD* | 7.83 | 0 |
| MCHK_8175 | *nifD* | 12.82 | 7.32E-10 | MCHK_8170 | *mhr8170* | 7.73 | 0 |
| MCHK_8174 | *nifK* | 12.23 | 5.05E-07 | MCHK_8182 | *mhr8182* | 7.66 | 0 |
| MCHK_6267 | *livG* | 12.18 | 4.25E-06 | MCHK_8226 | *nifB* | 7.62 | 0 |
| MCHK_8172 | *nifE* | 12.07 | 2.32E-05 | MCHK_8171 | *nifN* | 7.58 | 0 |
| MCHK_8169 | *nifX* | 11.72 | 5.60E-05 | MCHK_8173 | *mhr8173* | 7.58 | 0 |
| MCHK_8170 | *mhr8170* | 10.60 | 1.94E-04 | MCHK_8217 | *fixA* | 7.41 | 0 |
| MCHK_8171 | *nifN* | 10.58 | 9.92E-09 | MCHK_8183 | *mhr8183* | 7.41 | 0 |
| MCHK_8225 | *mhl8225* | 10.53 | 1.65E-04 | MCHK_8218 | *fixB* | 7.29 | 0 |
| MCHK_8217 | *fixA* | 10.44 | 1.16E-08 | MCHK_8219 | *fixC* | 7.26 | 0 |
| MCHK_8226 | *nifB* | 10.42 | 2.92E-06 | MCHK_8169 | *nifX* | 7.20 | 0 |
| MCHK_8218 | *fixB* | 9.95 | 5.37E-09 | MCHK_0868 | *mhr0868* | 7.15 | 0 |
| MCHK_8182 | *mhr8182* | 9.62 | 1.20E-04 | MCHK_8221 | *mhr8221* | 7.08 | 0 |
| MCHK_8227 | *mhl8227* | 9.50 | 1.96E-04 | MCHK_8227 | *mhl8227* | 6.72 | 0 |
| MCHK_8219 | *fixC* | 9.44 | 2.38E-12 | MCHK_8220 | *fixX* | 6.71 | 0 |
| MCHK_8173 | *mhr8173* | 9.44 | 2.90E-04 | MCHK_8184 | *mhr8184* | 6.69 | 0 |
| MCHK_8228 | *nifZ* | 9.30 | 6.31E-05 | MCHK_7135 | *mhr7135* | 6.30 | 0 |
| MCHK_7192 | *mhr7192* | 9.22 | 3.88E-05 | MCHK_7131 | *yoaF* | 6.27 | 0 |
| MCHK_8229 | *mhl8229* | 8.94 | 7.93E-05 | MCHK_7130 | *mhl7130* | 6.24 | 0 |
| MCHK_8184 | *mhr8184* | 8.94 | 2.90E-04 | MCHK_7129 | *mhl7129* | 6.18 | 0 |
| MCHK_8183 | *mhr8183* | 8.63 | 7.93E-05 | MCHK_2453 | *mhl2453* | 6.11 | 0 |
| MCHK_7139 | *mhl7139* | 8.35 | 2.53E-06 | MCHK_8222 | *mhr8222* | 6.00 | 0 |
