# Supplementary material for: RNA-Seq and Microarrays Analyses Reveal Global Differential Transcriptomes of Mesorhizobium huakuii 7653R between Bacteroids and Free-Living Cells
Source: PLoS One. 2014 Apr 2;9(4):e93626. doi: 10.1371/journal.pone.0093626 (PMC3973600; doi:10.1371/journal.pone.0093626)
Supplement: Table S4 — Top 25 down-regulated genes in M. huakuii 7653R bacteroids revealed by RNA-Seq and Microarrays. (DOC) [file pone.0093626.s008.doc]

**Table S4. Top 25 down-regulated genes in *M. huakuii* 7653R bacteroids revealed by RNA-Seq and Microarrays**

| **RNA-Seq** | | | | Microarrays | | | |
| --- | --- | --- | --- | --- | --- | --- | --- |
| GeneID | Gene | log2 Ratio | P-value | GeneID | Gene | log2 Ratio | Q-value (%) |
| MCHK_4452 | *mhl4452* | -20.84 | 8.73E-146 | MCHK_5400 | *mhr5400* | -5.51 | 0 |
| MCHK_1168 | *mhl1168* | -18.91 | 9.51E-42 | MCHK_5402 | *mhr5402* | -5.41 | 0 |
| MCHK_5400 | *mhr5400* | -17.88 | 4.76E-16 | MCHK_8256 | *mhl8256* | -5.35 | 0 |
| MCHK_0008 | *mhl0008* | -16.83 | 2.44E-39 | MCHK_5403 | *mhr5403* | -5.33 | 0 |
| MCHK_3454 | *mhl3454* | -16.58 | 8.90E-07 | MCHK_5401 | *mhr5401* | -5.09 | 0 |
| MCHK_2302 | *oppF* | -16.56 | 8.00E-52 | MCHK_4451 | *fliC* | -4.98 | 0 |
| MCHK_0010 | *pilQ* | -15.86 | 6.23E-14 | MCHK_0050 | *mhr0050* | -4.95 | 0 |
| MCHK_4454 | *mhl4454* | -15.72 | 1.31E-05 | MCHK_1367 | *mhr1367* | -4.74 | 0 |
| MCHK_2895 | *mhl2895* | -15.64 | 2.13E-12 | MCHK_6095 | *mhl6095* | -4.64 | 0 |
| MCHK_3024 | *mhl3024* | -15.60 | 2.61E-08 | MCHK_3007 | *mhl3007* | -4.64 | 0 |
| MCHK_1113 | *divJ* | -15.46 | 1.35E-44 | MCHK_1371 | *mhr1371* | -4.60 | 0 |
| MCHK_3025 | *mhl3025* | -15.42 | 2.44E-06 | MCHK_3006 | *mhl3006* | -4.46 | 0 |
| MCHK_5791 | *mhl5791* | -15.21 | 3.20E-04 | MCHK_3672 | *mhr3672* | -4.45 | 0 |
| MCHK_4778 | *phnL* | -15.13 | 4.45E-14 | MCHK_4452 | *mhl4452* | -4.42 | 0 |
| MCHK_3174 | *murB* | -15.12 | 1.63E-04 | MCHK_2306 | *gsiB* | -4.40 | 0 |
| MCHK_5966 | *artM1* | -15.11 | 5.54E-13 | MCHK_3728 | *nirV* | -4.33 | 0 |
| MCHK_2163 | *mhr2163* | -14.65 | 5.95E-05 | MCHK_2376 | *mhl2376* | -4.32 | 0 |
| MCHK_4779 | *phnX* | -14.58 | 1.33E-08 | MCHK_5216 | *mhl5216* | -4.29 | 0 |
| MCHK_5736 | *mhr5736* | -14.41 | 1.00E-07 | MCHK_1117 | *mhr1117* | -4.28 | 0 |
| MCHK_3238 | *mhl3238* | -14.38 | 9.85E-05 | MCHK_3673 | *mhr3673* | -4.27 | 0 |
| MCHK_3248 | *mhl3248* | -14.32 | 2.89E-06 | MCHK_0012 | *mhr0012* | -4.25 | 0 |
| MCHK_4914 | *mhl4914* | -14.24 | 2.28E-04 | MCHK_1058 | *mhl1058* | -4.24 | 0 |
| MCHK_6042 | *mhr6042* | -14.08 | 3.25E-07 | MCHK_1798 | *mhl1798* | -4.20 | 0 |
| MCHK_6029 | *hpoE* | -14.07 | 1.66E-07 | MCHK_8255 | *mhl8255* | -4.17 | 0 |
| MCHK_3696 | *mhl3696* | -13.91 | 1.05E-06 | MCHK_3342 | *ssuA1* | -4.16 | 0 |
